# The psychological distress and coping styles in the early stages of the 2019 coronavirus disease (COVID-19) epidemic in the general mainland Chinese population: A web-based survey

**DOI:** 10.1371/journal.pone.0233410

**Published:** 2020-05-14

**Authors:** Huiyao Wang, Qian Xia, Zhenzhen Xiong, Zhixiong Li, Weiyi Xiang, Yiwen Yuan, Yaya Liu, Zhe Li

**Affiliations:** 1 The Mental Health Center and National Clinical Research Center for Geriatrics, West China Hospital, Sichuan University, Chengdu, Sichuan, China; 2 School of Nursing, Chengdu Medical College, Chengdu, Sichuan, China; 3 The Third Department of Clinical Psychology, Karamay Municipal People’s Hospital, Karamay, Xinjiang, China; 4 The West China College of Medicine, Sichuan University, Chengdu, Sichuan, China; 5 Zun Yi Psychiatric Hospital, Zunyi, Guizhou, China; Chiba Daigaku, JAPAN

## Abstract

As the epidemic outbreak of 2019 coronavirus disease (COVID-19), general population may experience psychological distress. Evidence has suggested that negative coping styles may be related to subsequent mental illness. Therefore, we investigate the general population’s psychological distress and coping styles in the early stages of the COVID-19 outbreak. A cross-sectional battery of surveys was conducted from February 1–4, 2020. The Kessler 6 psychological distress scale, the simplified coping style questionnaire and a general information questionnaire were administered on-line to a convenience sample of 1599 in China. A multiple linear regression analysis was performed to identify the influence factors of psychological distress. General population’s psychological distress were significant differences based on age, marriage, epidemic contact characteristics, concern with media reports, and perceived impacts of the epidemic outbreak (all *p* <0.001) except gender (*p* = 0.316). The population with younger age (*F* = 102.04), unmarried (*t* = 15.28), with history of visiting Wuhan in the past month (*t* = -40.86), with history of epidemics occurring in the community (*t* = -10.25), more concern with media reports (*F* = 21.84), perceived more impacts of the epidemic outbreak (changes over living situations, *F* = 331.71; emotional control, *F* = 1863.07; epidemic-related dreams, *F* = 1642.78) and negative coping style (*t* = 37.41) had higher level of psychological distress. Multivariate analysis found that marriage, epidemic contact characteristics, perceived impacts of the epidemic and coping style were the influence factors of psychological distress (all *p* <0.001). Epidemic of COVID-19 caused high level of psychological distress. The general mainland Chinese population with unmarried, history of visiting Wuhan in the past month, perceived more impacts of the epidemic and negative coping style had higher level of psychological distress in the early stages of COVID-19 epidemic. Psychological interventions should be implemented early, especially for those general population with such characteristics.

## Introduction

The epidemic of the 2019 coronavirus disease (COVID-19) has aroused widespread concern throughout society in China. Because the virus can be transmitted through droplets, contact, etc. [1], cities in many regions of China have closed non-essential public places, restricted mass gathering activities, and enacted other control measures to effectively control the spread of the virus [2]. The epidemic has had a strong impact on general population’s daily life. At the same time, as the epidemic continues, general population gradually experience different levels of psychological distress, such as nervousness, fear of infection, anxiety, depression, sleep problems, and inattention [3,4]. Previous studies have reported that some psychological problems often occur during similar epidemic [5,6] or other traumatic stress events, such as natural disasters [7,8], disease [9], or long-term employment in high stress occupations [10–12], and may last for a long time [13,14].

When faced with stress or traumatic experiences, general population often responds differently, with some responding positively and others responding negatively. Evidence has suggested that coping styles in the face of stress have an impact on the quality of general population’s life [15,16], and negative coping styles may be related to psychological distress or mental illness such as post-traumatic stress disorder (PTSD), anxiety, and depression [7,8,12]. For this reason, we conducted this study in the early stages of this epidemic to investigate the general population’s psychological distress and coping style related to the epidemic of COVID-19 so that those who have high levels of psychological distress and/or respond negatively can be detected early and undergo timely intervention.

## Methods

This study was conducted through an online survey, starting at 16:00 on February 1, 2020 and ending at 24:00 on February 4, and the survey was approved by the ethical review board of the West China Hospital of Sichuan University. The snowball sampling method was used to invite subjects. All invitees completed the questionnaire online via Questionnaire Star (https://www.wjx.cn). An initial set of invitees (10 participants) was chosen to ensure broad representation of age, gender, occupation, education level, and city. This set of invitees then forwarded the questionnaire to 10 companions whom they considered suitable for the survey, and this second set forwarded the questionnaire in the same way. The study included a general population aged 18 years or older who volunteered to participate in the study. The participants received a complete description of this survey and were asked to sign an online informed consent prior to data collection. Respondents were excluded if they reported a history of mental illness and/or could not complete the online survey independently.

### Data collection

A self-made questionnaire was used to collect demographic and epidemiological information of participants, including gender, age, marriage, epidemic contact and concern characteristics, and perceived epidemic impacts of the epidemic of COVID-19.

### Psychological distress assessment

The Kessler 6 psychological distress Scale (K6) was used to assess the psychological distress of participants; this scale has been proven to have cross-cultural reliability and validity [17]. It contains six questions that ask participants to rate how often they have felt ‘nervous’, ‘hopeless’, ‘restless or fidgety’, ‘so depressed that nothing could cheer you up’, ‘that everything was an effort’, and ‘worthless’ during the past 30 days.

### Coping style assessment

The Simplified Coping Style Questionnaire (SCSQ) was used to assess the participants’ coping styles during the COVID-19 epidemic; this questionnaire has been proven to have good reliability and validity in Chinese [18]. The SCSQ contains twenty items, with each item using a four-point score (0 = never, 1 = seldom, 2 = often, 3 = always), and two subscales: positive coping (12 items) and negative coping (8 items). According to the average and standard deviation of the positive coping style and the negative coping style scores, a Z conversion is used to calculate their respective standard scores, and then, the negative coping standard scores are subtracted from the positive coping style standard scores to calculate the tendency value of coping style. A result greater than 0 was defined as a participant adopting a positive coping style when faced with stress, and a result less than 0 was defined as a participant adopting a negative coping style [19].

### Statistical analysis

Differences in psychological distress (K6 score) among categorical variables were tested by t-tests or one-way analysis of variance. A stepwise multiple linear regression analysis was performed to identify the influence factors of psychological distress. All statistical analyses were conducted in SPSS version 22.0 (IBM, Chicago, IL, USA), and p<0.05 was considered to be statistically significant.

### Quality control

The same IP address could be used only once to complete the questionnaire, which did not collect any personal information such as names, thereby ensuring anonymity and honest responses. The time spent on each questionnaire was monitored automatically, and the whole questionnaires completed in fewer than 120 seconds were rejected as invalid.

## Results

### Sample characteristics

There were 1607 individuals from 26 regions of China who completed this survey, and 1599 (99.5%) were included in the analysis participants. Among all participants, 1068 (66.8%) were female, 531 (33.2%) were male; ages ranged from 18 to 84 years old (mean 33.9±12.3 years); 914 (57.2%) were married, 685 were unmarried (42.8%); 326 (20.4%) had a history of visiting Wuhan; and 333 (20.8%) had a history of epidemics occurring in their community; 1583 (99.0%) concern with media reports related to the epidemic; 911 (56.9%) feel nervous, 767 (48.0%) feel difficult to control emotion and 612 (38.3%) have epidemic-related dreams in perceived impacts of the epidemic; 547(34.2%) respond with negative coping styles (Table 1).

**Table 1:** Sample description.

Variables	n (%)
**Total**	1599 (100.0)
**Demographic characteristics**	
Gender	
Female	1068 (66.8)
Male	531 (33.2)
Age (mean 33.9±12.3, years)	
18–30	722 (45.2)
31–40	471 (29.5)
41–50	254 (15.9)
>50	152 (9.5)
Marriage	
Unmarried	685 (42.8)
Married	914 (57.2)
**Epidemic contact characteristics**	
History of visiting Wuhan	
No	1273 (79.6)
Yes	326 (20.4)
History of epidemics occurring in the community	
No	1266 (79.2)
Yes	333 (20.8)
**Concern with media reports related to the epidemic**	
less concerned	16 (1.0)
Concerned	141 (8.8)
more concerned	428 (26.8)
extremely concerned	1014 (63.4)
**Perceived impacts of the epidemic**	
Changes over living situations	
feel relax	209 (13.1)
no change	479 (30.0)
feel nervous	911 (56.9)
Emotional control	
no difficult	832 (52.0)
less difficult	304 (19.0)
Difficult	70 (4.4)
more difficult	70 (4.4)
extremely difficult	323 (20.2)
Epidemic-related dreams	
No	987 (61.7)
Less	151 (9.4)
General	115 (7.2)
More	33 (2.1)
extremely large	313 (19.6)
**Coping style**	
Negative	547 (34.2)
Positive	1052 (65.8)

### Comparison of the psychological distress

The results revealed significant differences in the participants’ psychological distress based on age (*F* = 102.04), marriage (*t* = 15.28), history of visiting Wuhan or not (*t* = -40.86), history of epidemics occurring in the community or not (*t* = -10.25), concern with media reports related to the epidemic (*F* = 21.84), and changes over living situations (*F* = 331.71), emotional control (*F* = 1863.07), and epidemic-related dreams (*F* = 1642.78) in perceived impacts of the epidemic (all *p* <0.001); there were no significant differences based on gender (*t* = -1.00, *p* = 0.316). As age increases and marital status changes, K6 scores have a downward trend. Those with a history of visiting Wuhan and a history of epidemics occurring in the community have higher level of psychological distress than those without such experiences. The psychological distress tended to increase with concern with media reports related to the epidemic and perceived impacts of the epidemic. At the same time, the results also show that those with negative coping style have higher level of psychological distress than those with positive coping style (*t* = 37.41, *p* <0.001) (Table 2).

**Table 2:** Psychological distress (K6 scores) of participants (n = 1599).

Variables	Means (SD)	95%CI	t/F*	*p*
**Total**	7.7 (7.7)	7.36, 8.11		
**Demographic characteristics**				
Gender			-1.002	0.316
Female	7.6 (7.5)	7.15, 8.05		
Male	8.0 (8.1)	7.32, 8.69		
Age category (mean33.9±12.3, years)			102.04	<0.001
18–30	11.1 (8.9)	10.47, 11.76		
31–40	5.2 (5.3)	4.77, 5.73		
41–50	4.8 (4.7)	4.22, 5.36		
>50	4.3 (4.8)	3.55, 5.08		
Marriage			15.28	<0.001
unmarried	10.9 (9.0)	10.22, 11.57		
married	5.4 (5.4)	5.01, 5.72		
**Epidemic contact characteristics**				
History of visiting Wuhan			-40.86	<0.001
No	5.0 (4.8)	4.70, 5.23		
Yes	18.6 (7.1)	17.80, 19.34		
History of epidemics occurring in the community			-10.25	<0.001
No	6.8 (7.0)	6.37, 7.14		
Yes	11.5 (8.8)	10.50, 12.40		
**Concern with media reports related to the epidemic**			21.84	<0.001
less concerned	2.4 (2.6)	1.10, 3.65		
concerned	4.0 (3.9)	3.34, 4.62		
more concerned	6.9 (7.3)	6.19, 7.58		
extremely concerned	8.7 (8.0)	8.21, 9.20		
**Perceived impacts of the epidemic**				
Changes over living situations			331.71	<0.001
feel relax	2.4 (2.8)	2.01, 2.78		
no change	3.2 (3.0)	2.94, 3.47		
feel nervous	11.3 (8.2)	10.81, 11.87		
Emotional control			1863.07	<0.001
no difficult	3.0 (2.8)	2.84, 3.22		
less difficult	5.6 (3.5)	5.23, 6.01		
difficult	8.9 (3.9)	7.97, 9.80		
more difficult	10.3 (5.1)	9.11, 11.49		
extremely difficult	21.0 (3.3)	20.68, 21.39		
Epidemic-related dreams			1642.78	<0.001
No	3.6 (3.4)	3.38, 3.80		
Less	6.2 (3.8)	5.62, 6.83		
general	7.4 (4.5)	6.54, 8.18		
More	10.7 (4.6)	9.13, 12.27		
extremely large	21.4 (2.6)	21.06, 21.65		
**Coping style**			37.41	<0.001
negative	15.0 (8.3)	14.31, 15.70		
positive	4.0 (3.5)	3.75, 4.17		

* representing the differences of K6 scores among categorical variables by t-test or one-way analysis of variance

### Stepwise multiple linear regression analysis

Age, marriage, epidemic contact characteristics, perceived impacts of the epidemic and coping style were included in the multivariate analysis. Factors’ values were listed in Table 3. The results indicated that the model could explain 86.2% of the psychological distress (K6 scores) (R^2^ = 0.862, adjusted R^2^ = 0.862). Marriage (*β* = -0.588, *p*<0.001), history of visiting Wuhan (*β* = 1.449, *p*<0.001), changes over living situations (*β* = 1.040, *p*<0.001), emotional control (*β* = 1.995, *p*<0.001) and epidemic-related dreams (*β* = 1.597, *p*<0.001) in perceived impacts of the epidemic, and coping style (*β* = -2.135, *p*<0.001) were the influence factors of psychological distress (Table 4). Unmarried, history of visiting Wuhan, more serious changes over living situations, more difficult of emotional control, higher frequency of epidemic-related dreams, and negative coping style in the general population showed higher level of psychological distress.

**Table 3:** Factors’ values assigned in the stepwise multiple linear regression model.

Variables	Value
Age	Primary Value
Marriage	0 = Unmarried, 1 = Married
History of visiting Wuhan	0 = No, 1 = Yes
History of epidemics occurring in the community	0 = No, 1 = Yes
Concern with media reports related to the epidemic	Primary Value
Changes over living situations	Primary Value
Emotional control	Primary Value
Epidemic-related dreams	Primary Value
Coping style	0 = Negative, 1 = Positive

**Table 4:** Stepwise multiple linear regression analysis for psychological distress (K6 scores) of participants (n = 1599).

Variables	*β*	t	*p*	95%CI
Constant	3.526	13.939	<0.001	3.030–4.022
Marriage	-0.588	-3.682	<0.001	-0.901– -0.275
History of visiting Wuhan	1.449	5.265	<0.001	0.909–1.898
Changes over living situations	1.040	9.057	<0.001	0.815–1.266
Emotional control	1.995	21.879	<0.001	1.816–2.174
Epidemic-related dreams	1.597	16.964	<0.001	1.412–1.782
Coping style	-2.135	-10.575	<0.001	-2.531–-1.739

## Discussion

The results of the present study suggest that the general population in China mainland reported higher level of psychological distress in the early stages of COVID-19 than those in non-epidemic period [20]. The present study was conducted during the first two weeks of the COVID-19 outbreak, so it indicated that the general population have already presented psychological distress in the early stages of epidemic. This finding is consistent with the previous studies. The traumatic stress experiences during the occurrence of emergency events, such as major public events or natural disasters, were often related to the general population’s early psychological distress and subsequent mental illness [5,6,8]. The psychological distress could cause the impairment of individual’s normal daily activities and was associated with worse social functioning [21]. So, our result suggested that the psychological intervention for the general population should be provided urgently after the outbreak of COVID-19 epidemic.

The multivariate analysis showed that marriage was the influence factor of psychological distress in general population. The population with unmarried showed higher level of psychological distress. Unmarried status, lack of a major social support system, was found to be related to psychological distress [22]. The other study also found single mothers without additional personal support showed higher values of psychological distress [23]. These might imply the importance of the basic social support of marriage when an individual faced the COVID-19 epidemic and suggest that mental health workers should pay increased attention to unmarried population.

Our results also found that the history of visiting Wuhan in the past month was the influence factor of psychological distress. The population with history of visiting Wuhan showed higher level of psychological distress. Mandatory contact tracing and 14 days quarantine, which form part of the public health responses to the COVID-19 outbreak when having the history of visiting Wuhan in the past month. The quarantine may perpetuate the sense of danger and uncertainty and increase individuals’ psychological distress about the effects of contagion, infection, and stigma on their families and friends [3]. It indicated that the general population who have the history of visiting Wuhan should be paid more attention in the early stages of COVID-19 epidemic.

Perceived impacts on changes over living situations, emotional control, and epidemic-related dreams were found to be the influence factors of psychological distress. The population with more serious changes over living situations, more difficult of emotional control, and higher frequency of epidemic-related dreams showed higher level of psychological distress. Previous investigations on Ebola virus and severe acute respiratory syndrome (SARS) have found the similar phenomena that individuals might perceive the infectious diseases as a type of life-threatening life event which could cause various negative emotions, such as anxiety, depression, fear, and a series of sleep problems [3,24–27]. In addition, these symptoms may be risk factors for individuals suffering from mental illness in the future and affect individual’s attitudes and behaviors towards the epidemic prevention [25]. As of Jan 25, 2020, 30 Chinese provinces, municipalities, and autonomous regions have initiated first-level responses to major public health emergencies [3]. A range of measures has been urgently adopted, including closed non-essential public places, restricted mass gathering activities, delineating control areas, contact tracing and monitoring to effectively control the spread of the virus, which may bring public uncertainty and a sense of crisis [28, 29]. So, the sudden changes over living situations may increase individual’s anxiety and nervous. The sleep problems, especial recurrent distressing dreams in which the content are related to the traumatic event are the core symptoms in patients with PTSD [30]. So, this indicated that the general population may present the symptoms of PTSD in the early stages of COVID-19 epidemic. Difficult to control the emotion and emotional instability could lead to worsening psychological problems which have been reported in the outbreak of SARS [31]. Taken these results together, suggested that the psychological intervention should be implemented urgently for the general population who perceived more impacts on changes over living situations, emotional control, and epidemic-related dreams to prevent suffering from PTSD in the later time.

The multivariate analysis also showed that the coping style was the influence factor of psychological distress. The population with negative coping style showed higher level of psychological distress. The previous study related to traumatic stress events has reported that those in the general population with traumatic stress experiences were more likely to adopt a negative coping style [9]. Many previous studies have shown that different coping styles, especially negative coping styles, for trauma stress events are also related to subsequent mental illness [7,10,32]. In contrast, a positive coping style may promote emotional well-being [33]. Therefore, the general population with negative coping styles should be given attention and the appropriate psychological interventions should be considered urgently.

There are several limitations in our study. Firstly, the survey method is based on network invitation rather than face-to-face random sampling, and participants need to be able to use network tools. As a result, the status of the general population who cannot use network tools is unclear. Secondly, we did not assess whether and how respondents were engaging in prevention. Finally, our study design is cross-sectional and thus cannot capture changes in psychological distress and its predictors over the course of the COVID-19.

## Conclusion

Our study revealed that the epidemic of COVID-19 caused high level of psychological distress in the general population. The general mainland Chinese population with unmarried, history of visiting Wuhan in the past month, perceived more impacts of the epidemic and negative coping style showed higher level of psychological distress in the early stages of COVID-19 epidemic. Psychological interventions should be implemented urgently, especially for those in the general population with such characteristics.

## Supporting information

S1 Survey The psychological distress and coping style in the early stage of the 2019 coronavirus disease (COVID-19) epidemic questionnaire. (DOCX)

S1 Data (XLS)

S2 Data (XLSX)
