# Supplementary material for: The psychological distress and coping styles in the early stages of the 2019 coronavirus disease (COVID-19) epidemic in the general mainland Chinese population: A web-based survey
Source: PLoS One. 2020 May 14;15(5):e0233410. doi: 10.1371/journal.pone.0233410 (PMC7224553; doi:10.1371/journal.pone.0233410)
Supplement: S1 Survey — (DOCX) [file pone.0233410.s001.docx]

S1 Survey of the psychological distress and coping style in the early stage of the 2019 coronavirus disease (COVID-19) epidemic in the general mainland Chinese population

# The psychological distress and coping style in the early stage of the 2019 coronavirus disease (COVID-19) epidemic Questionnaire

**Demographics**

1. **Gender**

- Male
- Female

1. **Age**

**_____** years

1. **Marriage status**

- Married
- Unmarried

**Instructions: The following questions will ask about your information on the epidemic of COVID-19. Please select the answer of your choice.**

1. **Did you have a visit to Wuhan in the past month?**

- Yes
- No

1. **Was an epidemic occurring in your community in the past month?**

- Yes
- No

1. **Which of the following matches your concern with media reports related to the epidemic?**

- less concerned
- concerned
- more concerned
- extremely concerned

1. **Which of the following matches your perceived changes over living situations related to the epidemic?**

- feel relax
- no change
- feel nervous

1. **Which of the following matches your perceived impact over emotional control related to the epidemic?**

- no difficult
- less difficult
- difficult
- more difficult
- extremely difficult

1. **How much epidemic-related dreams do you have?**

- no
- less
- general
- more
- extremely large.

**K6: In the past month, how often did you feel: (Please select the corresponding option in each question that matches your situation)**

|  | No | Occasionally | Part of the time | Most of the time | All the time |
| --- | --- | --- | --- | --- | --- |
| 1. Nervous |  |  |  |  |  |
| 1. Hopeless |  |  |  |  |  |
| 1. Restless or fidgety |  |  |  |  |  |
| 1. So depressed that nothing could cheer you up |  |  |  |  |  |
| 1. Everything was an effort |  |  |  |  |  |
| 1. worthless |  |  |  |  |  |

**SCSQ: The following lists the attitudes and practices that you may take when you are hit by setbacks or encounter difficulties in your life during the epidemic. Please read each item carefully and then select the corresponding option in each question that matches your situation.**

|  | No use | Occasional use | sometimes use | frequent use |
| --- | --- | --- | --- | --- |
| 1. Relieved through work study or other activities |  |  |  |  |
| 1. Talk with others, express troubles |  |  |  |  |
| 1. Try to see the good side of things |  |  |  |  |
| 1. Change your mind and rediscover what is important in life |  |  |  |  |
| 1. Don't look the problem too seriously |  |  |  |  |
| 1. Stick to your own position and fight for what you want |  |  |  |  |
| 1. Identify different kinds of methods to solve problems |  |  |  |  |
| 1. Ask for advice from relatives, friends or classmates |  |  |  |  |
| 1. Change some of the original practices or some of your own problems |  |  |  |  |
| 1. Learn from others on handling similar difficult situations |  |  |  |  |
| 1. Cultivate hobbies and actively participate in cultural and sports activities |  |  |  |  |
| 1. Try to restrain your disappointment, regret, sadness and anger |  |  |  |  |
| 1. Attempt to take a break or vacation, and temporarily abandon the problem (trouble) |  |  |  |  |
| 1. Relieve trouble by smoking, drinking, taking medicine and eating |  |  |  |  |
| 1. Believe that time will change the status, the only thing to do is to wait |  |  |  |  |
| 1. Trying to forget the whole thing |  |  |  |  |
| 1. Rely on others to solve problems |  |  |  |  |
| 1. Accept reality because there is no other way |  |  |  |  |
| 1. Imagine that one kind of miracle might happen and change the status |  |  |  |  |
| 1. Comfort yourself |  |  |  |  |

**Thank you for participating in this survey.**
